# Knowledge, Attitudes, and Perceptions of Artificial Intelligence in Healthcare Among Medical Students at Sultan Qaboos University

**DOI:** 10.7759/cureus.44887

**Published:** 2023-09-08

**Authors:** Zinah A Al Hadithy, Abdullah Al Lawati, Riham Al-Zadjali, Hamed Al Sinawi

**Affiliations:** 1 College of Medicine and Health Sciences, Sultan Qaboos University, Muscat, OMN; 2 General Practice, Sultan Qaboos University Hospital, Muscat, OMN; 3 Psychiatry and Behavioral Sciences, Sultan Qaboos University Hospital, Muscat, OMN

**Keywords:** medical school curricula, healthcare, artificial intelligence (ai), clinical year medical students, knowledge attitudes perception

## Abstract

Background

Artificial intelligence (AI) is increasingly used in healthcare, but more data are needed about the knowledge, perceptions, attitudes, and preparedness of medical students in Oman towards this technology. This study aimed to investigate these aspects among clinical-year medical students at Sultan Qaboos University.

Methodology

A web-based validated exploratory questionnaire adapted from a study conducted at the University of Toronto was distributed to all clinical year (phase III) medical students at Sultan Qaboos University. The questionnaire collected demographic and background information, tested students’ knowledge of AI, and assessed their perceptions and attitudes toward it. The data were analyzed using the Statistical Package for Social Sciences (SPSS, IBM Corp., Armonk, NY).

Results

A total of 221 out of 368 clinical-year medical students (60%) completed the survey. Most respondents were in their junior clerkship year (n = 94, 42.5%). Most students (n = 167, 75.4%) had no prior exposure to AI in healthcare, with a median knowledge score of 3.25 out of 5 in AI, and showed no improvement over the years. However, they overall had positive perceptions and attitudes towards AI. Students also had concerns about the impact of AI on employment prospects and ethical issues but were generally receptive to incorporating AI into medical school curricula, as 174 students (78.7%) believed every medical trainee should receive training on AI competencies.

Conclusion

This study provides valuable insights into the knowledge, perceptions, attitudes, and preparedness of medical students in Oman toward AI in healthcare. Medical educators in Oman should consider incorporating AI into medical school curricula to prepare future physicians for using this technology in healthcare.

## Introduction

Artificial intelligence (AI) in medicine refers to using computers and advanced technology, such as machine learning algorithms, to assemble and process data input from experts and analyze it, producing critical thinking comparable to that of a human being [1]. AI has been primarily employed for tasks involving visual imagery, where it can analyze images and detect any abnormal phenotypic characteristics in them to formulate a hypothesis regarding the patient's underlying condition [2]. An example is doing tasks such as recognizing tumors or examining X-rays [3]. The recent introduction of AI in healthcare, most popularly in radiology, has been a revolutionary advancement as it minimizes potential errors and maximizes efficiency [4]. Additionally, AI has been used successfully in predicting schizophrenia onset and improving existing laboratory testing methods in pathology [3,5]. In summary, medical AI can automatically detect pathologies in imaging examinations, assist in decision-making, and provide output that benefits doctors and patients.

The use of AI in medicine is rapidly growing in developed countries and has recently begun to be implemented in developing countries, with expectations for further growth [3,4]. For example, a study conducted in Ontario, Canada, found that most medical students had a positive attitude toward the aid of AI in diagnosis, with similar results found in a study conducted in Japan [6,7]. However, a multicenter study conducted in three major medical schools found that a small majority disagreed that AI could help make an automated diagnosis [2].

Moreover, developing countries like Pakistan still need to catch up in AI education and research, particularly in the healthcare field. A study conducted in Peshawar, Pakistan, showed that only a little over one-third of respondents had previous knowledge of AI [4]. Another study, also conducted in Pakistan, found that only 19.4% of the students had knowledge about AI in medical applications specifically [3].

Experts in AI have supported the notion that AI will impact all clinicians in the future [6]; therefore, there is a strong need for incorporating basic AI training into undergraduate and postgraduate medical curricula to compensate for these discrepancies. Moreover, there has been a growing acknowledgement that undergraduate medical education must play an important role in providing this background on the capabilities and limitations of using AI tools in medical practice [6]. While most student participants in the above-mentioned studies had a positive attitude towards introducing AI in undergraduate medical education, data on medical students' perceptions of AI still needs to be improved due to the emerging nature of AI and their general lack of knowledge about the topic [8]. Their understanding of AI and its medical applications may influence their attitudes toward artificial intelligence. The knowledge gap arises from inadequacies in the design of the medical curriculum to incorporate modern medical advances, including AI, which the already-overloaded curricula with limited room could cause for additional topics and a scarcity of faculty with the expertise to teach AI [1,9]. Nonetheless, AI concepts should be integrated into the pre-existing courses; this can help reduce the possible curricular overload and highlight its clinical applications. Efforts in this aspect have already been undertaken. For example, some medical schools have started offering AI courses, such as the Carle Illinois College of Medicine in the United States, Queen's University in the United Kingdom, and the University of Toronto in Canada [10].

It is important to mention that certain issues may arise with the introduction of AI-based systems, particularly in terms of privacy as well as data security. Therefore, it is crucial that the confidentiality of medical records is protected while introducing AI, as health records and data are extremely vulnerable [11]. Moreover, patient consent is a serious issue when it comes to data privacy, as the introduction of AI into the health system may aspire to multiple research projects on the topic and subsequently allow a wide usage of patient information without necessitating patient approval [12,13].

Therefore, as the need to incorporate these competencies into medical education rises, analyzing medical students' baseline knowledge, perceptions, and concerns regarding clinical AI may give more insight into key areas for curriculum development. There are currently no published studies concerning this in Oman. Therefore, this study aims to assess the knowledge, perceptions, attitudes, and preparedness of clinical-year medical students at Sultan Qaboos University in Oman towards introducing AI in healthcare and is thought to be the first study conducted in Oman on this topic.

## Materials and methods

Study design

This cross-sectional, self-administered, survey-based study was conducted by distributing a web-based questionnaire prepared using Google Forms.

Study population and sample size

The study population included all medical students in their clinical phase of undergraduate study, known as "phase III," studying at Sultan Qaboos University. This included pre-clerkship (first clinical year), junior-clerkship (second clinical year), and senior-clerkship (third clinical year) students. Medical students in their preclinical years, as well as non-medical students enrolled in healthcare programs, were excluded from the study. All students were sent an invitation to participate, with clear indications that participation was voluntary and anonymous. Formal consent was obtained from the participants. The invitation was disseminated through the student's university email, by the college administration, and through social media platforms.

Considering that the total number of clinical year students at SQU is 368 and using the sample size for the population mean formula at a confidence level of 95% and a margin of error of 5%, a total sample size of 189 respondents was required for this study. Our sample included 221 respondents, and they were all sampled randomly.

Data collection

A questionnaire was prepared by modifying questions taken from a previous Canadian study of the same topic, prepared by Mehta et al. [6], and adapting the questions to be more suitable for students in Oman. The questionnaire was completely in English and was made using Google Forms. Ethical approval was obtained from the Medical Research and Ethics Committee (MREC) at the College of Medicine and Health Sciences, Sultan Qaboos University. The questionnaire was validated through a pilot test initially among a small group before being disseminated to the whole sample.

Questionnaire information

The questionnaire comprised three main sections with a total of 49 questions.

The first section collected demographic and background information about the participants, such as gender, study year, and knowledge background. The second section included questions answered using the Likert scale. The scale used options ranging from 1, "strongly disagree," to 5, "strongly agree," with 3 being "neutral," to test the extent of familiarity of the students with important terms relating to AI in healthcare and thus their knowledge of AI. The third section of the study aimed to understand students' perceptions and attitudes toward AI. A definition was provided at the beginning to assist those unfamiliar with the terminology. The questions were categorized into sub-sections with specific themes. A Likert scale was utilized, offering options such as "extremely unlikely," "unlikely," "neutral," "likely," and "extremely likely."

The first sub-section asked students to estimate the possibility of AI replacing physicians for certain tasks, using adaptive questioning for the time frame based on the responses. The other parts of this section explored students' perspectives on the impact of AI on physician employment prospects, new ethical and societal issues, and the implementation of AI in medical school. The questionnaire concluded with an optional open-ended question, allowing students to share their thoughts on AI.

Data analysis

Data was analyzed using the Statistical Package for Social Sciences (SPSS) version 27.0 (released 2020; IBM Corp., Armonk, New York, United States). The median of the knowledge score and perception score were calculated. The Kruskal-Wallis test was used to compare the knowledge score across the three years of study and employed the Mann-Whitney U test to evaluate gender-based differences. P-values of <0.05 were considered statistically significant. However, most of the information provided in this work is descriptive and has been described using numbers and percentages.

## Results

A total of 228 medical students took part in the study. Seven participants were excluded because they provided incomplete answers and did not meet the inclusion criteria. This left us with 221 valid responses out of 368 students. The majority of respondents were junior clerkship students, comprising 42.5% of the participants, followed by pre-clerkship students at 31.7%. Regarding gender, 54.3% of respondents were female, while 45.7% were male. Interestingly, a significant portion of the participants had no academic background in computer science (62.4%) and had not attended any lectures or taken classes related to AI (80.5%) or programming and coding (83.3%).

Knowledge

**Figure 1:**
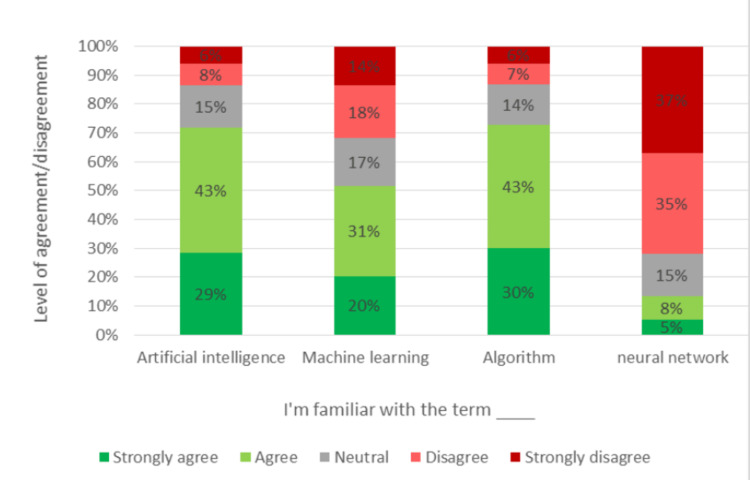
Questionnaire responses of students’ familiarity with artificial intelligence terms

Figure 1 illustrates the participants' understanding of various terms related to artificial intelligence. Among the participants, 71.9% stated that they understood the term "artificial intelligence." While 72.9% of them agreed or strongly agreed with knowing the term "algorithm," only 51.6% of the participants agreed or strongly agreed to be familiar with the term "machine learning." Surprisingly, only a small percentage of the participants, 13.5%, agreed or strongly agreed with knowing the term "neural network." The complete data are represented in Figure 1.

**Figure 2:**
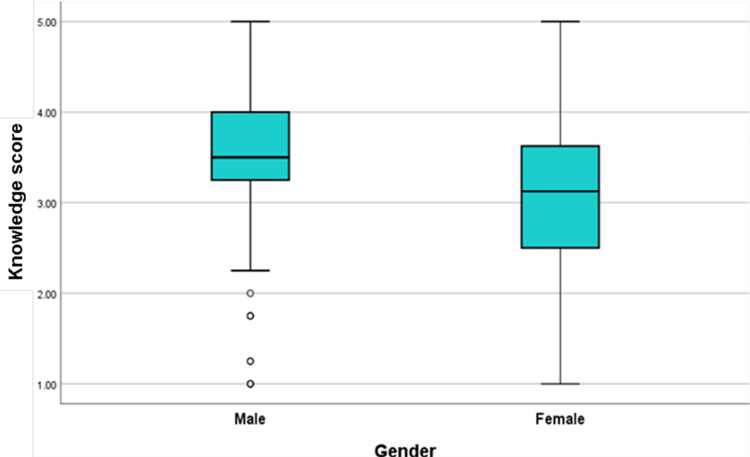
Questionnaire responses of students’ median knowledge stratified by gender

**Figure 3:**
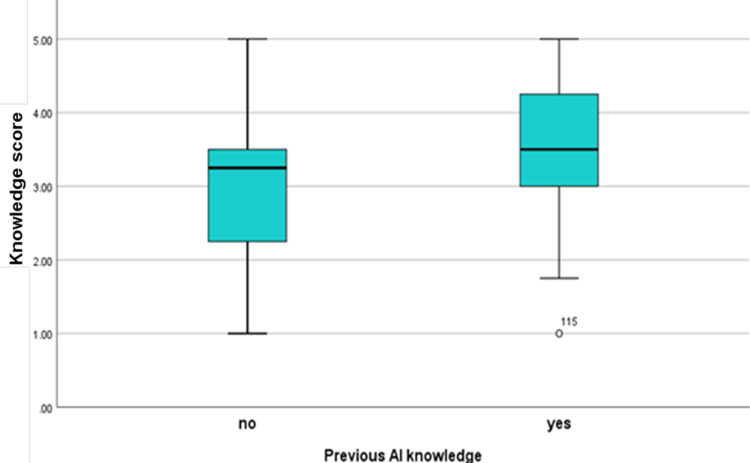
Questionnaire responses of students’ median knowledge stratified by previous AI knowledge

The knowledge score on the Likert scale (1 = strongly disagree to 5 = strongly agree) was calculated and found to have a median of 3.25 (1.00-5.00), and there were no statistically significant variations in the knowledge score across the various years of study (p=0.214). However, when the knowledge score was compared by gender, it was statistically significant (p=0.002). Similarly, when the association was tested between the knowledge score and having any academic background in AI, it was found to be statistically significant (p≤0.001), as illustrated in Figures 2-3.

Perceptions

**Figure 4:**
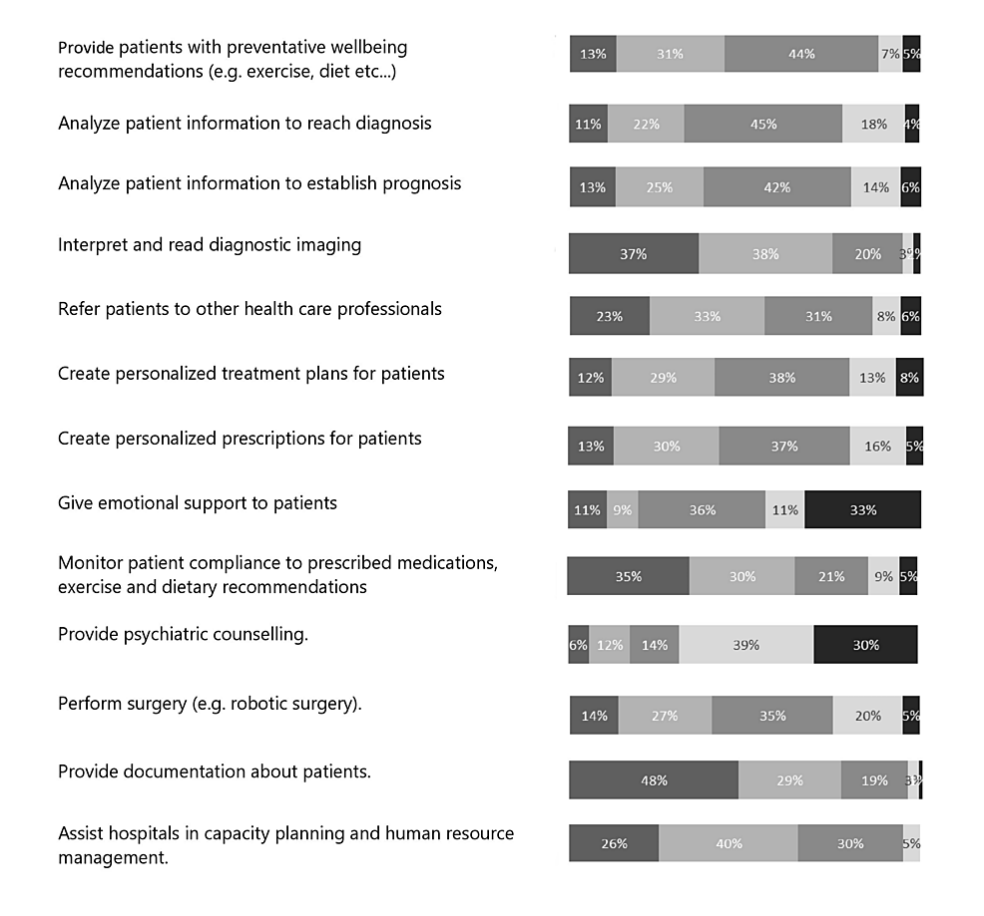
Perceptions regarding the timelines for when AI will the replace the performance of the listed tasks Left to right: 0-4 years, 5-10 years, 11-25 years, 26-50 years, more than 50 years

The students were asked about their perceptions of the likelihood of AI technologies replacing or excelling healthcare professionals in certain tasks, divided into two main themes: individual patient care and population health. The average perception score on the Likert scale (1 = extremely unlikely to 5 = extremely likely) was 3.2. The complete dataset is presented in Table 1, and the perceptions regarding the timeline for these capabilities to be achieved are presented in Figure 4.

**Table 1:** Likert scale questionnaire responses of students’ perceptions on the likelihood of artificial intelligence replacing medical specialties AI: artificial intelligence ^‡^In your opinion, what is the likelihood that artificial intelligence technologies will be able to replace/excel the average healthcare professional in performing the following tasks?

AI will replace the task of a physician to^‡^	Extremely unlikely	Unlikely	Neutral	Likely	Extremely likely
Provide patients with preventative well-being recommendations (e.g., exercise, diet, etc.).	4.5% (10)	19.0% (42)	19.0% (42)	41.2% (91)	16.3% (36)
Analyze patient information to reach a diagnosis	3.6% (8)	16.3% (36)	16.3% (36)	42.5% (94)	21.3% (47)
Analyze patient information to establish prognosis	2.3% (5)	15.4% (34)	23.5% (52)	44.8% (99)	14.0% (31)
Interpret and read diagnostic imaging	3.2% (7)	9.0% (20)	11.8% (26)	24.4% (54)	51.6% (114)
Refer patients to other healthcare professionals	9.0% (20)	37.6% (83)	14.9% (33)	22.2% (49)	16.3% (36)
Create personalized treatment plans for patients	11.3% (25)	28.5% (63)	23.1% (51)	27.6% (61)	9.5% (21)
Create personalized prescriptions for patients	9.5% (21)	28.5% (63)	19.5% (43)	33.5% (74)	9.0% (20)
Give emotional support to patients	69.2% (153)	14.0% (31)	5.4% (12)	8.1% (18)	3.2% (7)
Monitor patient compliance to prescribed medications, exercise, and dietary recommendations	19.5% (43)	24.0% (53)	16.3% (36)	25.3% (56)	14.9% (33)
Provide psychiatric counseling	60.6% (134)	21.7% (48)	6.3% (14)	10.0% (22)	1.4% (3)
Perform surgery (e.g., robotic surgery)	10.4% (23)	16.3% (36)	11.3% (25)	38.5% (85)	23.5% (52)
Provide documentation about patients	1.8% (4)	8.1% (18)	10.9% (24)	30.8% (68)	48.4% (107)
Assist hospitals in capacity planning and human resource management	5.4% (12)	10.0% (22)	17.6% (39)	40.7% (90)	26.2% (58)

Written below is a summary of the responses that showed the greatest disparity

When it came to the ability to reach a diagnosis and establish a prognosis, the majority of respondents (n=94, 42.5% and n=99, 44.8%, respectively) thought it was likely, with most believing that AI would replace healthcare professionals within 11-25 years (n=66, 44.6% and n=59, 42.4%, respectively). In regards to reading diagnostic imaging, an even larger majority (n=114, 51.6%) believed that it was extremely likely or likely (n=54, 24.4%) to be replaced by AI, with most thinking that it would happen within 5-10 years (n=64, 37.9%), followed by within 0-4 years from now (n=63, 37.3%). On the contrary, the great majority of respondents (n=153, 69.2%) believed it was extremely unlikely for AI to replace healthcare staff in providing emotional support, followed by (n=31, 14.0%) who thought it was unlikely. Similarly, most respondents (n=134, 60.6%) believed it was extremely unlikely for AI to excel in providing psychiatric counseling, and (n=48, 21.7%) thought it was unlikely. However, concerning performing robotic surgery, a greater majority of respondents stated that it is likely (n=85, 38.5%) followed by extremely likely (n=52, 23.5%) that AI would have the potential to replace the average healthcare professional in the coming years, which most anticipated would be within 11-25 years (n=50, 34.7%).

Attitudes

The question of whether AI will reduce the number of job opportunities available to physicians has elicited a range of responses. A small minority of those surveyed (n=54, 24.4%) expressed disagreement, while a larger majority expressed agreement (n=135, 61.1%) that AI would diminish the number of job positions available to physicians. The remaining (n=32, 14.5%) were neutral about the topic. Furthermore, respondents also believed that certain medical specialties are more susceptible to job loss due to AI, with a majority agreeing (n=97, 43.9%) or strongly agreeing (n=75, 33.9%) and the minority disagreeing (n=15, 6.8%) or strongly disagreeing (n=9, 4.1%).

**Figure 5:**
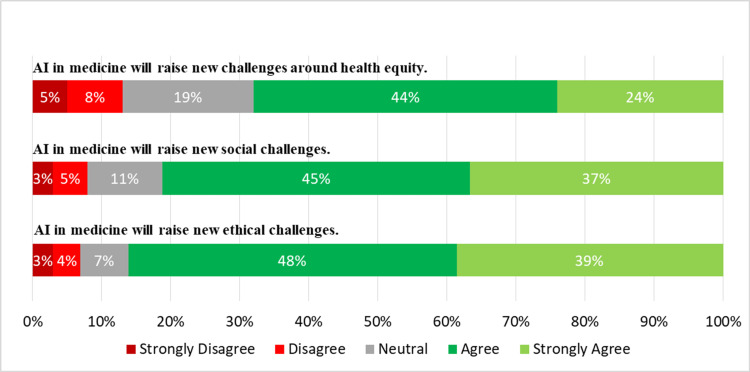
Extent of agreement among students to the challenges posed by AI

Additionally, the students were questioned about their level of agreement with some statements concerning the involvement of AI in ethical, social, and health equity challenges. Figure 5 illustrates the extent of agreement among students about the challenges posed by AI.

**Figure 6:**
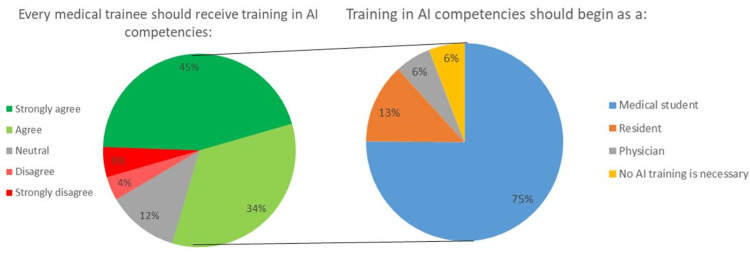
Questionnaire responses of students’ attitudes toward using artificial intelligence

Concerning AI and education, many respondents stated that the Omani healthcare system needs to be adequately prepared to deal with AI-related difficulties. More specifically, 52.9% of students (n=117) strongly disagreed, and 23.5% (n=52) disagreed with the statement, "The Omani healthcare system is currently well-prepared to deal with challenges having to do with AI." Respondents also expressed concerns regarding their medical education's sufficiency in preparing them to work alongside AI tools, as (n=107, 48.4%) strongly disagreed and (n=53, 23.9%) disagreed with the statement "My medical education is adequately preparing me for working alongside AI tools." This question showed no statistically significant differences between the different study years of phase III (p=0.214). Additionally, most respondents strongly believed (n=109, 49.3%) or believed (n=67, 30.3%) that medical training should include training on AI competencies. A significant proportion of respondents (n=174, 78.4%) also believed that every medical trainee should be required to receive training in AI competencies, and (n=166, 75.1 %) felt that training should begin as a medical student, followed by (n=29, 13.1%) as a resident. More details are available in Figure 6.

## Discussion

Knowledge

This study aimed to explore medical students' knowledge, perceptions, and attitudes regarding the integration of AI in the healthcare sector. The study sample primarily comprised junior clerkship students, most of whom lacked formal education in AI. The majority of participants were familiar with the terms "artificial intelligence" and "algorithm," and approximately half with "machine learning," while only a small subset was familiar with the term "neural network," which is considered a fundamental concept in deep learning, a subfield of AI. These results suggest that students have some basic knowledge of AI but need a more in-depth understanding. A similar trend was seen in a study conducted in Nepal, Pakistan, and Saudi Arabia [4,10,14]. Recent research has also explored medical students' attitudes and knowledge of AI in healthcare, where medical students had a positive outlook toward AI in healthcare but needed to gain significant knowledge on the subject [15]. This result is in line with the findings of the current study. To ensure that future healthcare providers have a thorough understanding of this new, growing technology, it is crucial to prioritize AI education in the medical curriculum. Notably, there was no significant disparity in average knowledge scores between different academic years. However, gender and a prior background in AI were significantly associated with variations in the average knowledge score. The same was reported in another study [2]. This tells us that giving lectures on AI and so on impacts the student’s overall knowledge.

Perception

The findings of this study provide insight into medical students' perceptions of the potential impact of AI on healthcare. Overall, students' perceptions of AI technologies replacing or excelling healthcare workers in specific jobs were moderate, with an average score of 3.2 out of 5.0. This implies that medical students recognize the potential of AI in healthcare but are skeptical of its ability to replace healthcare workers in certain jobs. The study also found that students believed AI technology had the potential to improve healthcare overall, and they believed that AI could assist healthcare professionals in tasks such as analyzing patient information to reach a diagnosis and prognosis, interpreting and reading diagnostic imaging, and performing surgery. These findings are consistent with the results of other studies that have shown that AI algorithms can achieve higher accuracy and efficiency in certain clinical tasks compared to healthcare professionals, particularly in tasks requiring large data processing [16]. Interestingly, the participants' perceptions varied widely regarding the potential for AI to replace physicians in referring patients to other healthcare professionals and creating personalized treatment plans and prescriptions for patients. This may be because such tasks require a combination of clinical judgment, empathy, and communication skills, which are challenging to replicate through AI technologies. The findings further suggest that AI may have a role in assisting clinicians in these tasks rather than fully replacing them. In contrast, the respondents believed it was extremely unlikely for AI to replace healthcare workers in tasks such as giving emotional support and providing psychiatric counseling. This suggests that medical students recognize the importance of the human touch in certain areas of healthcare, where empathy and emotional intelligence are essential components of care. The study also investigated the students' perceptions regarding the timeline for AI technologies to replace or excel healthcare professionals in certain tasks, which varied widely. The results showed that the students believed that AI would unlikely replace healthcare professionals in most tasks in the very near future. However, they believed that tasks such as providing documentation about patients, monitoring patients' compliance with prescribed medication, and interpreting diagnostic imaging could be taken over by AI as soon as within the next four years or the next 5-10 years for the task of hospital capacity planning. This finding agrees with previous studies that have suggested that AI is most likely to take over healthcare workers in tasks that include data analysis [16].

Attitudes

The findings of this study suggest that medical students generally perceive AI as a technology that has the potential to impact healthcare professionals' employment prospects, with a majority of respondents agreeing or strongly agreeing that AI will lessen the number of jobs available to physicians. This perception aligns with previous studies investigating healthcare professionals' attitudes toward AI in the workplace. Zhang et al. reported that healthcare professionals were concerned about job loss due to AI and perceived AI as a technology that would replace humans in certain tasks [17]. Similarly, a study by Leslie-Mazwi and Lev found that using AI in healthcare could reduce certain medical specialties' workforce [8]. Furthermore, the respondents in this study also believed that certain medical specialties were more susceptible to job loss due to AI than other specialties. This perception is consistent with the findings of a study by Ahmed et al., which reported that healthcare professionals perceived radiology and pathology as the medical specialties most likely to be impacted by AI, as they concluded that the potential influence of AI on these specializations is because they involve the interpretation of medical imagery, a job that AI can accomplish with accuracy and speed [3]. However, the impact of AI on healthcare occupations is yet unknown, and there are possible prospects for new positions and duties to arise as a consequence of AI deployment [8]. In addition to concerns about job loss, most respondents in this survey agreed or strongly agreed that AI in medicine would present new ethical, social, and health equity challenges. Moreover, the findings of the research reveal that medical students in Oman have worries about the readiness of the healthcare system to cope with AI-related difficulties and the sufficiency of their medical education in preparing them to work alongside AI technologies. Most respondents also agreed that medical training should include training on AI competencies, suggesting that they're prepared to adapt and accept the role of AI in healthcare. This finding is consistent with the growing recognition of the importance of AI education in healthcare, as AI is increasingly used in clinical decision-making and patient care [8]. The fact that a large majority of respondents stated that every medical trainee should be obliged to undergo training in AI capabilities indicates the necessity for an integrated curriculum on AI education in medical institutions.

Limitations and future directions

When interpreting the findings, it is important to consider the current study's limitations. To begin with, the study is conducted on clinical-year medical students from Sultan Qaboos University in Oman. This means that the study's results might not be generalizable to other populations, other universities, or to medical students in their earlier years of study. Moreover, the study is based on a self-administered questionnaire. As with any self-reported data, there's a potential for bias, including recall bias, social desirability bias (participants might provide answers they think are acceptable rather than their true feelings or knowledge), or misunderstanding questions. Although the study provides a glimpse into the students' understanding of various AI-related terms, it does not delve deep into how well these terms are understood or how proficient the students are in these areas. Out of 228 medical students who took part in the study, 7 were excluded due to incomplete answers. This raises the issue of non-response bias, where those who chose not to complete the survey might have different views or levels of knowledge compared to those who did. In addition, the study compares data with previous studies conducted in other countries like Canada, but there might be significant cultural, educational, or socio-economic differences that could influence the results. It is important to keep in mind the limitations of the language barrier, as the questionnaire was entirely in English. While medical education in many countries is conducted in English, language might still pose a barrier to complete understanding or articulation of opinions for some students. Furthermore, the students' understanding of AI-related terms was tested with specific terms like "algorithm," "machine learning," and "neural network." However, the world of AI is vast, and there might be many more relevant terms and concepts that the students are unfamiliar with. While the study did use an open-ended question to gather qualitative data, the majority of the data is quantitative. This might not capture the nuance or depth of the student's understanding or perceptions regarding AI. Lastly, a significant portion of the participants had no academic background in computer science and had not attended any lectures or taken classes related to AI or programming. Their perceptions of AI might be influenced by this lack of exposure, and the study might not capture the views of those who have a more informed background in the subject. Therefore, future studies should aim to replicate these findings in larger and more diverse samples to understand regional variations in AI knowledge and attitudes.

## Conclusions

This research highlights that medical students at Sultan Qaboos University possess basic knowledge of AI but need a more in-depth understanding, indicating a need for greater AI education in the medical curriculum. While they recognize AI's potential to enhance healthcare, they remain cautious about its ability to replace human skills in certain tasks and have concerns about job prospects and ethical implications. Further studies with broader samples from different institutions are needed to make it more generalizable in Oman.
